# Children’s Recall of Fast Food Television Advertising—Testing the Adequacy of Food Marketing Regulation

**DOI:** 10.1371/journal.pone.0119300

**Published:** 2015-03-04

**Authors:** Amy M. Bernhardt, Cara Wilking, Diane Gilbert-Diamond, Jennifer A. Emond, James D. Sargent

**Affiliations:** 1 Norris Cotton Cancer Center, Geisel School of Medicine at Dartmouth, Lebanon, New Hampshire, United States of America; 2 Public Health Advocacy Institute, Northeastern University School of Law, Boston, Massachusetts, United States of America; 3 Department of Community and Family Medicine, Geisel School of Medicine at Dartmouth, Lebanon, New Hampshire, United States of America; 4 Department of Pediatrics, Geisel School of Medicine at Dartmouth, Lebanon, New Hampshire, United States of America

## Abstract

**Background and Aim:**

In the United States, the fast food companies McDonald’s and Burger King participate in marketing self-regulation programs that aim to limit emphasis on premiums and promote emphasis of healthy food choices. We determine what children recall from fast food television advertisements aired by these companies.

**Methods:**

One hundred children aged 3–7 years were shown McDonald’s and Burger King children’s (MDC & BKC) and adult (MDA & BKA) meal ads, randomly drawn from ads that aired on national US television from 2010–11. Immediately after seeing the ad, children were asked to recall what they had seen and transcripts evaluated for descriptors of food, healthy food (apples or milk), and premiums/tie-ins.

**Results:**

Premiums/tie-ins were common in children’s but rarely appeared in adult ads, and all children’s ads contained images of healthy foods (apples and milk). Participants were significantly less likely to recall any food after viewing the children’s vs. the adult ad (MDC 32% [95% confidence interval 23, 41] vs. MDA 68% [59, 77]) p <0.001; BKC 46% [39, 56] vs. BKA 67% [58, 76] respectively, p = 0.002). For children’s ads alone and for both restaurants, recall frequency for all food was not significantly different from premium/tie-ins, and participants were significantly more likely to recall other food items than apples or milk. Moreover, premiums/tie-ins were recalled much more frequently than healthy food (MDC 45% [35, 55] vs. 9% [3, 15] p<0.001; BKC 54% [44, 64] vs. 2% [0, 5] respectively, p<0.001).

**Conclusions:**

Children’s net impressions of television fast food advertising indicate that industry self-regulation failed to achieve a de-emphasis on toy premiums and tie-ins and did not adequately communicate healthy menu choices. The methods devised for this study could be used to monitor and better regulate advertising patterns of practice.

## Introduction

In 2009, spending on food marketing to youth in the United States was $1.79 billion, with $1 billion directed at children aged 2–11 years [1]. Television was the predominant medium to reach youth, and quick service or fast food restaurants spent $154 million on television advertising aimed at the child demographic. Fast food restaurants spent more than $487 million on cross-promotion activities that included licensing fees and marketing related to promoting premiums and tie-ins; an additional $341 million was spent to distribute the premiums [1]. In light of these expenditures, the Federal Trade Commission (FTC) concluded, “[c]ross-promotion was the hallmark of marketing food to young people, particularly children [1].” There is increasing concern that food advertising shapes the way children eat and contributes to childhood obesity [2]. Children view an average of 13 food-related advertisements on television per day, and there is experimental evidence that viewing them increases energy intake, both temporarily [3,4] and in the long-term [5]. To assess children’s television fast food advertising, we previously evaluated a one-year sample (July 2009-June 2010) of nationally televised fast food ads from the top 25 fast food companies [13] and found that 99% of children’s ads were from just two companies, McDonald's (70%) and Burger King (29%).

Although food marketing to children is subject to state and federal consumer protection laws, these laws are rarely enforced in the United States; instead, the advertisements are monitored for fairness and deceptive practices through industry self-regulation. McDonald’s and Burger King are members of the Children’s Food and Beverage Advertising Initiative (CFBAI) and the Children’s Advertising Review Unit (CARU), both administered by the Council of Better Business Bureaus. Companies participating in CFBAI pledge to market foods and beverages that meet certain nutritional criteria in children’s advertising [6]. In 2010 McDonald’s and Burger King had agreed to show meals containing their healthier side options of apples and milk with a standard hamburger or chicken option [7,8].

CARU maintains a set of guidelines that govern how all products are marketed to children [9]. CARU guidelines state that marketing should not be deceptive, and that deception is determined by gauging the net-impression of an entire advertisement on the target audience of children. CARU explicitly addresses toy premiums: “[s]ince children have difficulty distinguishing product from premium, advertising that contains a premium message should focus the child's attention primarily on the product and make the premium message clearly secondary [9].” Over the years, McDonald’s and Burger King have been cited by CARU for violating its premium guideline [10] and agreed to take CARU recommendations into account in future ads featuring premiums. When a member company does not adequately remedy an issue cited in a CARU complaint, CARU can refer the case on to the Federal Trade Commission [11,12].

Our content analysis of McDonald’s and Burger King children’s television advertisements revealed that children’s ads placed little emphasis on the food, and toy premiums and tie-ins were presented prominently in the visual and audio elements [13]. In contrast, the adult ads emphasized the food—its taste, portion size, and price. We concluded that the companies did not follow through with their self-regulatory promises. The Better Business Bureaus responded to the publication of these results with the following statements, “[b]oth CFBAI and CARU extensively monitor food advertising directed to children for compliance with each program,” and “[t]he content of the entire ad and net impression [among children] guide CARU’s determinations [of whether the ad violates self-regulatory standards] [14].”

In this study, we obtained the net impression of children ages 3–7 years to children’s and adult advertisements from McDonald’s (for the Happy Meal) and Burger King (for the BK Kids Meal). At the time of the study, children’s meals were marketed to children under age 12 according to the 2007 CFBAI pledges of both companies [15,16]. We chose to focus on children ages 3–7 years because cognitive research indicates that young children cannot effectively recognize the persuasive intent of advertising or apply the critical evaluation required to comprehend commercial messages [17]. In light of these limitations, CARU requires premium messages be clearly secondary to food messages. Therefore, we compared recall of premiums and food after seeing the children’s ads. We also determined whether recall of food included any of the healthy items (all the children’s ads contained images of apples and milk) that the companies have pledged to promote through CFBAI. Finally, since developmental limitations might also hamper the likelihood young children will report food, even after seeing it, we compared their response to food in children’s compared to adult ads, where we had previously found food images to be more salient [13].

## Methods

### Study sample

Children aged 3–7 were identified through an electronic medical record search from a general pediatrics clinic serving a Northern New England rural population. We obtained a partial HIPAA waiver from the Dartmouth Committee for Protection of Human Subjects to allow the transfer of a partial dataset from the clinic to the research setting. Prior to record transfer, a clinic secretary mailed a letter from the clinic physicians to all eligible families briefly describing the project, endorsing it, and including a phone number that could be called if any parent objected to being contacted by the researchers. Fewer than 10 (< 3%) of parents called to request that their information not be transferred, and those records were removed prior to transfer of contact information.

We recruited by telephone, using insurance status information to enrich the sample, so about 40 percent of the children would be from Medicaid-insured families. We succeeded in contacting 217 parents of age-eligible children, of whom 133 agreed to participate. Four children were excluded because the child did not verbalize a response to any ad, six were excluded due to technical failures of the videotaping equipment, and 23 failed to show after repeated attempts to schedule. After exclusions and failures to show, our final sample size was 100, for a participation rate of 46 percent. All aspects of the research were approved by the Committee for the Protection of Human Subjects at Dartmouth.

### Food ads sample

We obtained all food ads from McDonald’s and Burger King that aired nationally between July 2010 and June 2011 (n = 258) from a media surveillance company (http://www.kantarmedia.com). Ads were reviewed to determine whether they advertised children’s meals (McDonald’s Happy Meals or BK Kids’ Meals) or adult meals, and whether the material was appropriate for children in our age group. The majority of ads were 30 seconds in duration; 103 ads were eliminated to make ad length comparable across all four categories. Ads were then re-examined, and 33 were eliminated because they duplicated material found in other retained ads.

Some of the 30-second McDonald’s children’s meal ads consisted of two sequential 15-second spots; these were shown sequentially with no break in between. Five of the McDonald’s children’s meal ads were eliminated because they were directed at parents. Two McDonald’s and eight Burger King adult ads were deleted because the material was deemed inappropriate for children as young as 3 years of age. One of the authors (AB) screened the ads and then showed potentially problematic ones to the mother of a 5 year old. If the mother registered any concern, the ad was deleted from the ad pool. This left us with 30 McDonald’s children’s meal (MDC) ads, 44 McDonald’s adult meal (MDA) ads, 13 Burger King children’s meal (BKC) ads and 20 Burger King adult meal (BKA) ads for the television advertisements pool. None of the adult meal ads contained a premium message. All of the children’s meal ads, except 5 of the McDonald’s children’s ads, contained a premium message.

### Response assessment

Children were scheduled to come to the research laboratory with one parent. Written informed consent was obtained from a parent or guardian on behalf of the minor/child prior to starting the study. While the parent completed a questionnaire, the child was shown one of each of the four types of ads, with each one randomly selected from the ad pool and shown in random order, with the procedure carried out by an internally developed software program that also displayed the ads to the children.

We told the children that they were going to see a “television story” and we wanted them to explain what they saw in each one. We used the term television story because many children in this age range do not understand the term advertisement [17]. After seeing each ad, the child’s net impression was determined by asking, “What was that story about?” In parallel with other studies of advertising recall in children [18,19], participants were prompted to continue talking until they had nothing more to say about the commercial.

Responses were videotaped and the audio portion of the tape was transcribed. We illustrate this approach with videotapes of three children, each responding to one children’s and one adult advertisement. S1 and S2 Videos show children in the younger part of the age distribution, and S3 Video depicts an older child. Parents of these children were informed of the terms of the PLOS open-access license, viewed the videos, and provided written permission for publication under the terms of this license (see “Consent Form for Publication in a PLOS Journal”).

Transcripts of the children’s responses were coded into the following categories: food item, healthy food item (subset of food item involving recall of apples or milk), and premium/tie-in (recall of a premium or toy or a movie or television show). Two independent researchers transcribed all audio recordings. Any disagreement on the categorization of a word response was resolved by discussing with a third party.

### Statistical analysis

Children’s responses, defined as one or more words versus no mention per each category, were dichotomized to reflect any recall or no recall of food, healthy food (i.e., apples or milk), or premium/tie-ins. The proportion of children with any recall for each of the three categories was summarized by ad type, and McNemar’s test was used to determine if the likelihood of any recall differed by ad type. Report of premiums was not compared by type of ad because adult ads rarely use premiums to promote food. Results are presented for McDonald’s and Burger King restaurants, separately. All analyses were completed with the R Language and Environment for Statistical Computing, version 3.0.1 [20].

## Results

### Study sample description

The 100 participants ranged from 3 to 7 years of age, with an equal number of boys and girls (Table 1). Approximately half of families were covered by private insurance and 37% by Medicaid. There was wide variability in the mother’s educational attainment, with 17% of mothers having an education level of high school or less and 23% of mothers with an advanced graduate degree. There was also wide variability in household income, with 13% of participants from families with annual income <$30,000 and 28% of participants from families with annual income >$100,000.

**Table 1:** Characteristics of the children enrolled in the study.

Characteristic		N
Age		
	3	14
	4	20
	5	19
	6	21
	7	26
Gender		
	Male	50
	Female	50
Race/ethnicity		
	White, Non-Hispanic	85
	Black, Non-Hispanic	2
	Hispanic	3
	Asian	5
	Multi-race, Non-Hispanic	5
Health insurance		
	Private	57
	Medicaid	37
	Self-pay	6
Home ownership		
	Owns home	64
	Rents	29
	Other	7
Annual household income		
	<20K	7
	20–29K	6
	30–49K	23
	50–74K	15
	75–100K	15
	>100K	28
	Preferred not to answer	6
Mother’s education		
	High school diploma or less	17
	Some college	19
	Associate’s or Bachelor’s degree	38
	Master’s degree	15
	Doctoral or medical degree	8

Total sample size N = 100; values reflect N and percentages.

### Food and premium/tie-in recalls

**Fig 1:**
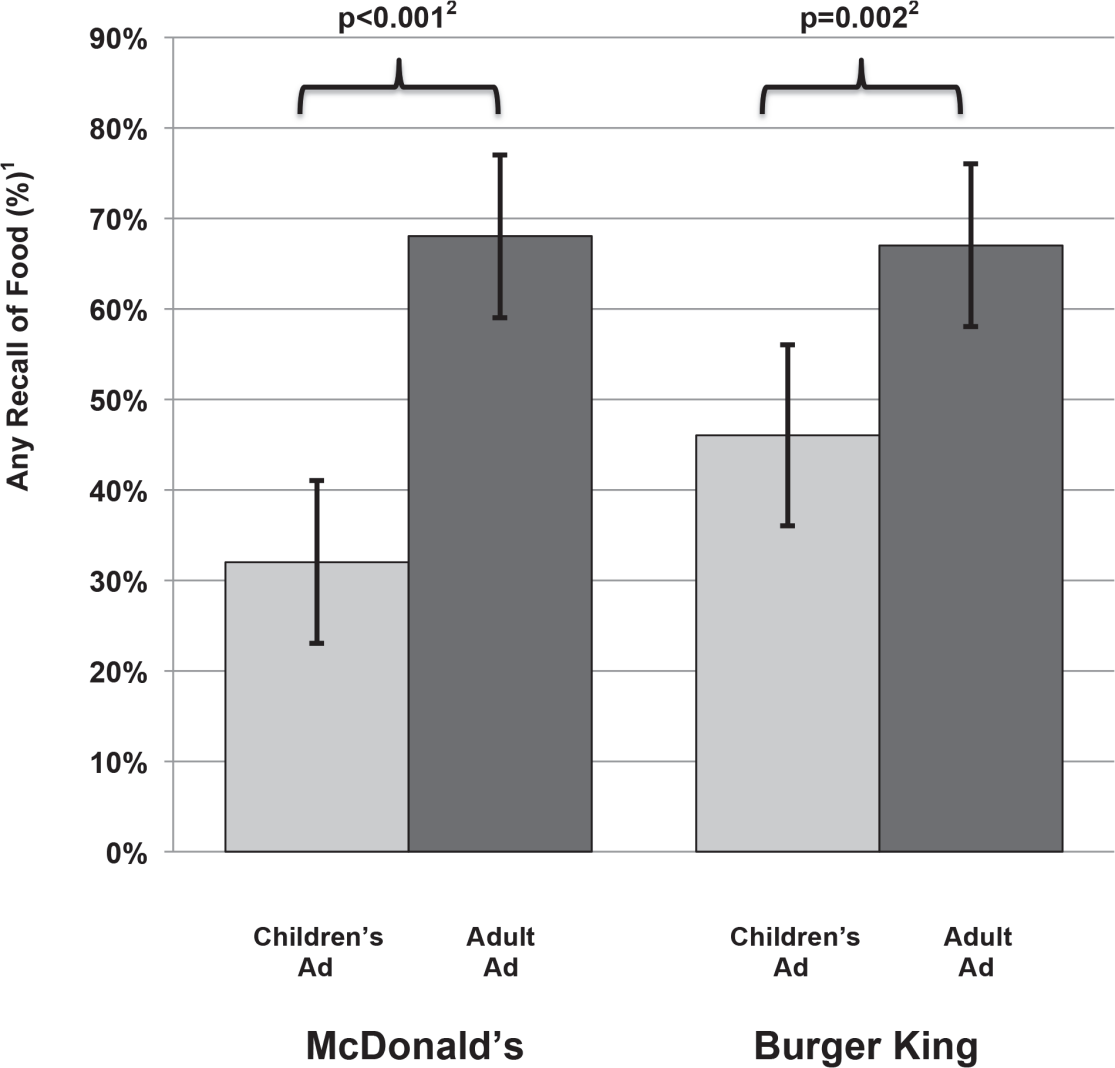
The proportion of children with any recall of food after seeing fast-food television advertising, by company (McDonalds, Burger King) and ad type (children’s, adult). Any recall of food presented as the proportion of all children (N = 100) who used one or more food related word when describing an ad; proportions are presented with 95% confidence intervals. For each restaurant, any recall of food was compared by ad type with McNemar’s Chi-Square test to account for the repeated measures on each child.

Participants used a median of 13 words (interquartile range: 5–23) when describing the children’s ads, and 9 words (3–22) when describing the adult ads. Fig. 1 shows the percent of participants with any recall of food by company and whether the ad targeted children or adults. For both companies, participants were significantly less likely to recall food after viewing the children’s ad compared to after the adult ad (MDC 32% [95% confidence interval: 23, 41] vs. MDA 68% [95% CI: 59, 77], McNemar’s Chi-square p <0.001; BKC% 46% [95% CI: 39, 56] vs. BKA 67% [95% CI: 58, 76], p = 0.002).

**Fig 2:**
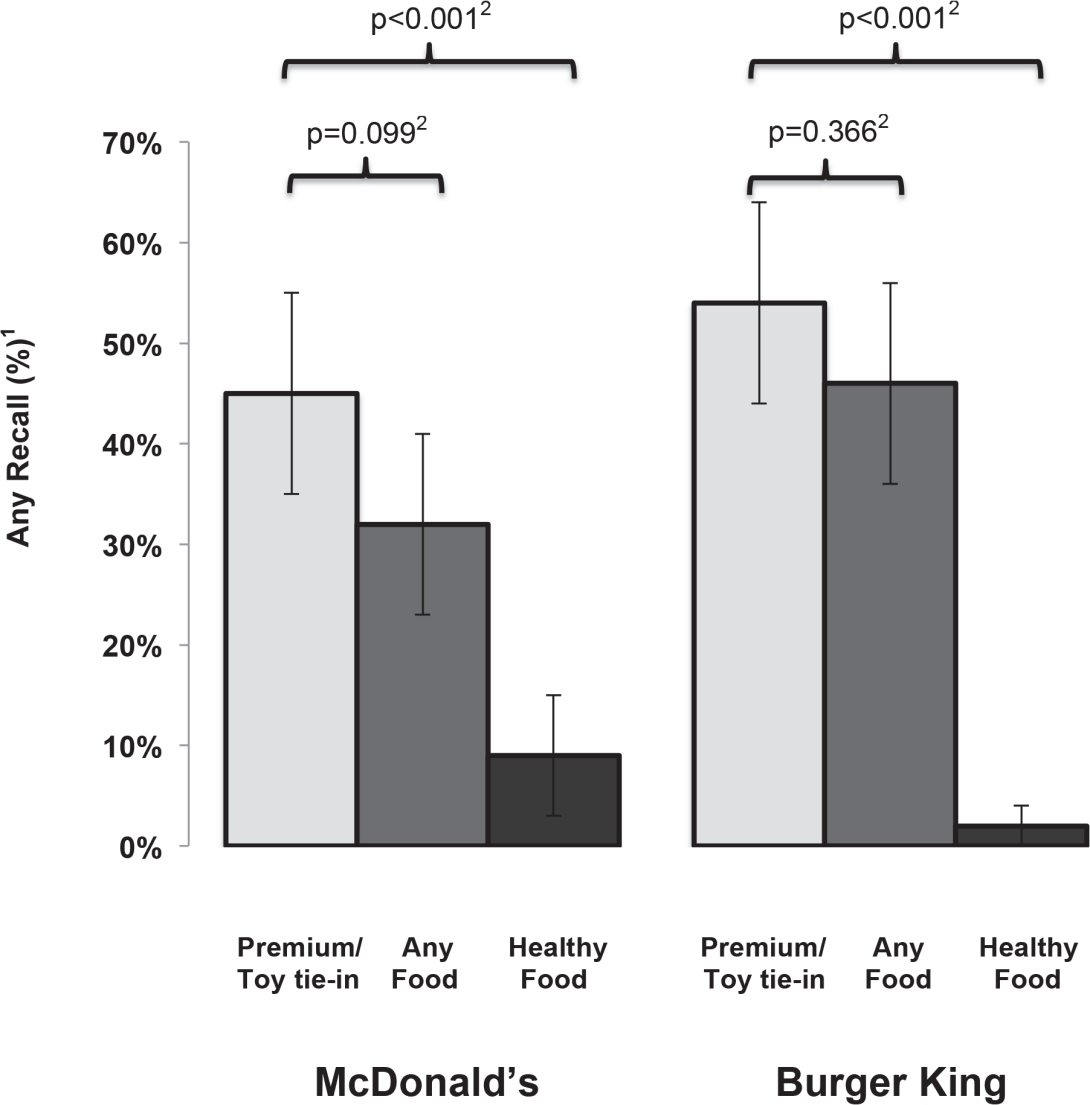
The proportion of children with any recall of 1) a premium/tie-in, 2) any food, or 3) healthy food after seeing fast-food television advertising targeted to children, by company (McDonalds, Burger King). Any recall of food presented as the proportion of all children (N = 100) who used one or more food related word when describing an ad; proportions are presented with 95% confidence intervals. For each restaurant, any recall of food was compared by ad type with McNemar’s Chi-Square test to account for the repeated measures on each child.

Fig. 2 focuses on ads for children’s meals only, comparing any recall of food or healthy food to any recall of premium/tie-in. Any recall of a premium/tie-in did not differ by restaurant (p = 0.223). Any recall of a premium/tie-in was higher than food (but the differences not statistically significant) for both companies (e.g., for MDC, food 32% [95% CI: 23, 41], toy/tie-in 45% [95% CI: 35, 55], p = 0.099). When participants did notice food, their recall rarely included mention of apples or milk, and any recall of healthy food was much lower than any mention of a premium/tie-in (MDC 9% [95% CI: 3, 15] vs. 45% [95% CI: 35, 55] respectively, p<0.001; BKC 2% [95% CI 0, 5] vs. 54% [95% CI: 44, 64] respectively, p<0.001). Any recall of healthy food was also much lower than for "less healthy food" (MDC 30% [95% CI: 21, 39], p<0.001 compared to healthy food; BKC 46% (95% CI: 36, 58), p<0.001 compared to healthy food). Results stratified by age (age 3–5 [n = 53] and age 6–7 [n = 47]) were consistent with the overall findings with no interaction.

## Discussion

This study gauged the net impression of children’s fast food televised advertising on the intended audience. We found that two thirds of children shown MDC ads and half shown BKC ads failed to recall any food at all and were just as likely to recall premiums, even though the advertisements were supposed to emphasize food and make premiums secondary. When children did recall food, they rarely mentioned the healthy choices these companies purported to be advertising under CFBAI, even though such images were included in all of the children’s ads. In contrast, most children recalled food after watching adult fast food advertisements, which shows that they had the developmental capability of noticing food when it is the primary focus of the advertisement. These findings are consistent with those of a recently published content analysis that assessed television ads from both companies and concluded that there was an under-emphasis of food in children’s ads compared to adult ads [13].

The fast food ads shown to the children in this study were conceived, produced and nationally televised during a time when: 1) expenditures on food marketing to children was the subject of a major FTC study; 2) an Inter-Agency Working Group created by Congress was formulating voluntary nutrition standards for foods marketed to children; 3) these companies made public CFBAI pledges to market healthy menu options; and 4) CARU guidelines specifically addressed the use of toy premiums in children’s advertising. Nonetheless, after seeing these ads, children rarely recalled healthy food options and recalled toy premiums/movie tie-ins as often as food, which would seem to violate CARU guidelines on premiums. We further conclude that depictions of healthy menu options, while present, were not salient, and that McDonald’s and Burger King failed to place appropriate emphasis on the food they sell in ads aimed at children. In response, they should enhance the visual elements of healthy food options by giving these images more time and greater size, and by including mentions of the healthy choices in the audio track—an acknowledgment that many of the children targeted by these advertisements cannot read. This was rarely done in children’s fast food advertising from these companies during the study period. For example, a content analysis conducted one year after companies made CFBAI pledges found that MDC and BKC fast food ads mentioned apples and milk in the audiotrack only 10 and 1 percent of the time respectively [13].

There is a larger problem with self-regulation as it is currently implemented—it has little or no impact on the ad in violation of the code because the ad has typically stopped running by the time the case is finalized. We propose an alternate approach: to formulate enforcement actions based on a “pattern or practice of marketing.” The pattern or practice approach looks at an overall course of conduct and crafts sanctions to address the practice in all future marketing. This study offers a novel way to assess children’s responses in a pattern or practice context. First, identify all children’s ads that aired nationally for a certain period. Second, randomly assign them to a sample of children in the target audience to determine what the children recall.

In the context of the present study, there are two issues to address—the over emphasis of premiums, and the under emphasis of healthy food choices. We interpret CARU guidelines, which state “advertising that contains a premium message should focus the child's attention primarily on the product and make the premium message clearly secondary,” to imply that children’s recall of premiums should be *significantly lower* than reports of food. Therefore, the fact that children shown a random sample of McDonald’s and Burger King children’s ads were as likely to recall a toy premium/movie tie-in message as any food at all demonstrates a pattern of practice by these companies that should be remediated. The findings also call into question the statement by CFBAI and CARU representatives that they were extensively monitoring food advertising for compliance with their programs during the study period.

Future evaluations of CARU and CFBAI self-regulation compliance should take a pattern or practice approach and consider not just whether ads contain images of foods meeting specified nutrition criteria, but also whether children actually identify them as such. Regulators at the FTC could mandate self-regulatory bodies to establish appropriate patterns of conduct to achieve the desired emphasis on healthy food and a de-emphasis on premiums. For example, a guideline might state that children must recall a healthy food option at least 80 percent of the time and a premium/movie tie-in less than 20 percent after seeing children’s fast food ads.

Beyond regulatory reforms on children’s advertising, there are simple practices that may be recommended to parents. Food advertising is ubiquitous on network and cable television; an average hour includes 11 food ads, and the overwhelming majority are for high-calorie, low nutrient food products that should not be part of a regular diet [21]. More than half of these advertising dollars are aimed at children under 12 years and airs on popular channels like Nickelodeon and Cartoon Network. Parents who wish to limit exposure to television marketing aimed at young children have many options in today’s multi-media environment. Parents wishing to stick to network programming could reduce exposure by blocking channels, like Nickelodeon, that refuse to comply with minimal nutritional standards for food advertising [22]. Those who wish to go further and eliminate all exposure to advertising may subscribe to video-on-demand services that omit traditional commercials (e.g., Netflix, Amazon, I-Tunes, etc.). Given the concern about childhood obesity and its relation with highly advertised energy dense foods, teachers and child health providers should be familiar with these alternatives and how to access them.

Media researchers can help by conducting interventional trials that explore whether switching to commercial-free media affects eating, finding ways to motivate parents to select commercial-free media venues for their children and assisting them in making that transition. At this point in time, we know very little about the ecology of home media viewing or how to influence it. By limiting exposure to commercial advertising, and by altering the content of the entertainment children watch [23], home media interventions have enormous potential to alter children’s caloric consumption.

This study has many strengths and some limitations as well. The study is novel—we know of no previous study that has addressed fairness and perception of content according to CARU guidelines. Net impression (our measure of compliance) was measured with little bias by allowing children to recall everything they remembered immediately after viewing an ad with no prompts or predefined response options. Identifying all ads shown during the study period and randomly assigning ads to children allowed us to make conclusions without bias that would occur in using only a subset of ads. Finally, assessing responses to adult ads from the same period ruled out developmental bias, as children frequently identified food in ads known to emphasize food [13]. Regarding limitations, our study lacks a measure of reliability and does not measure longer-term recall. The results may not apply to ads that are currently being aired, since the ad sample was drawn from between 2010 and 2011. The study was designed to provide a sample of children with wide socioeconomic but not race/ethnicity diversity, so the findings may be less applicable to minority children. Finally, this study did not attempt to make ad-by-ad comparisons of content with children’s recall, only to their net impression of children’s and adult ads from the time period.

In conclusion, this study assessed what children recalled after seeing McDonald’s and Burger King television advertising for fast food. Children were less likely to recall food after viewing children’s compared to adult fast food television ads, and few children recalled the healthy foods that both companies purported to be advertising in their children’s ads. Instead, they commonly recalled a premium or a movie character tie-in. Self-regulation of children’s fast food advertising aims to achieve a de-emphasis on premiums and to communicate healthy menu choices. The results of the present study raise concerns about self-regulation of children’s fast food advertising, along with the need to move toward pattern or practice assessments in the evaluation of all commercial advertising aimed at children.

## Supporting Information

S1 Video Study participant age 4 year 1 month responding to a Burger King adult television commercial and to a McDonald’s children’s television commercial. The video contains copyright footage from Burger King and McDonald’s advertisements, presented under fair use in order to illustrate the study methodology and differential response to adult compared to children’s advertisements.
(MP4)

S2 Video Study participant, 4 years 8 months responding to a Burger King adult television commercial and a Burger King children’s television commercial. The video contains copyright footage from Burger King advertisements, presented under fair use in order to illustrate the study methodology and differential response to adult compared to children’s advertisements.
(MP4)

S3 Video Study participant, 7 year 11 months responding to a Burger King adult television commercial and a Burger King children’s television commercial. The video contains copyright footage from Burger King advertisements, presented under fair use in order to illustrate the study methodology and differential response to adult compared to children’s advertisements.
(MP4)
